# Supplementary material for: Simultaneous Comparison of Subxiphoid and Intercostal Wound Pain in the Same Patients Following Thoracoscopic Surgery
Source: J Clin Med. 2022 Apr 18;11(8):2254. doi: 10.3390/jcm11082254 (PMC9030809; doi:10.3390/jcm11082254)
Supplement: Supplementary file 1 [file jcm-11-02254-s001.zip › Figure S1.pdf]

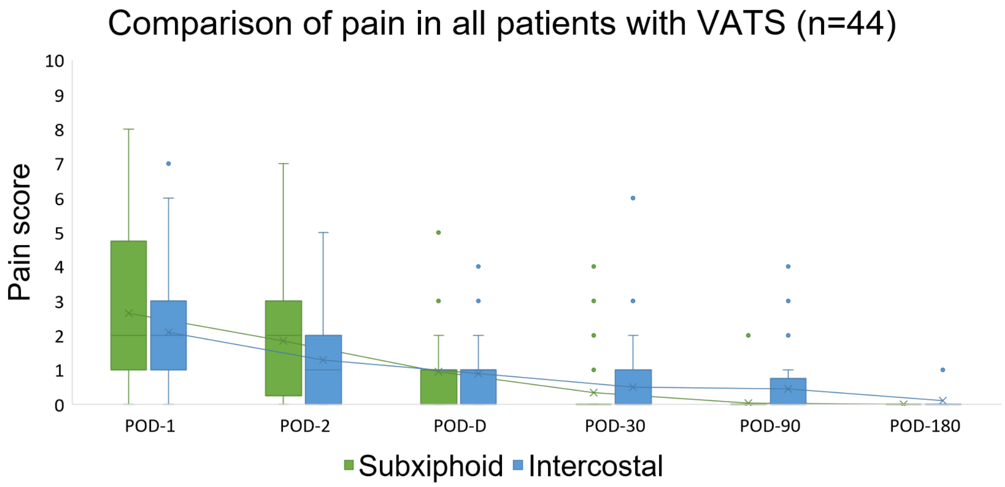

Figure S1: A box and whisker plot demonstrating pain score between subxiphoid and intercostal incisions for the same patient who received bilateral uniportal and unilateral multiportal.
