# Supplementary material for: Simultaneous Comparison of Subxiphoid and Intercostal Wound Pain in the Same Patients Following Thoracoscopic Surgery
Source: J Clin Med. 2022 Apr 18;11(8):2254. doi: 10.3390/jcm11082254 (PMC9030809; doi:10.3390/jcm11082254)
Supplement: Supplementary file 1 [file jcm-11-02254-s001.zip › Table S1.pdf]

Supplemental Table S1: Postoperative numerical rating scale (NRS) pain score for overall patients

Pain score	All VATS (n = 44)				
	Subxiphoid wound	95% CI	Intercostal wound	95% CI	P-value
POD-1	2.6 ± 2.0	(2.0 – 3.3)	2.1 ± 1.5	(1.6 – 2.6)	0.06
POD-2	1.8 ± 1.7	(1.3 – 2.3)	1.3 ± 1.3	(0.9 – 1.7)	0.01
POD-Discharge	1.0 ± 1.1	(0.6 – 1.3)	0.9 ± 0.9	(0.6 – 1.2)	0.62
POD-30	0.3 ± 0.8	(0.1 – 0.6)	0.5 ± 1.1	(0.2 – 0.8)	0.23
POD-90	0.1 ± 0.3	(0.0 – 0.1)	0.5 ± 1.0	(0.2 – 0.8)	0.008
POD-180	0 ± 0	(0.0 – 0.0)	0.1 ± 0.3	(0.0 – 0.2)	0.02

NRS: numerical rating scale; VATS: video-assisted thoracoscopic surgery; CI: confidence interval; POD: postoperative day; data were expressed as mean ± standard deviation
